# Nickel and Epigenetic Gene Silencing

**DOI:** 10.3390/genes4040583

**Published:** 2013-10-25

**Authors:** Hong Sun, Magdy Shamy, Max Costa

**Affiliations:** 1 Department of Environmental Medicine, NYU School of Medicine, Tuxedo, NY 10987, USA; E-Mail: Hong.Sun@nyumc.org; 2 Department of Environmental Sciences, Faculty of Meteorology, Environment and Arid Land Agriculture, King Abdulaziz University, Jeddah 21589, Saudi Arabia, E-Mail: Madgyshamy@hotmail.com

**Keywords:** epigenetics, gene silencing, heterochromatin, DNA methylation, histone modification, miRNA

## Abstract

Insoluble nickel compounds are well-established human carcinogens. Occupational exposure to these compounds leads to increased incidence of lung and nasal cancer in nickel refinery workers. Apart from its weak mutagenic activity and hypoxia mimicking effect there is mounting experimental evidence indicating that epigenetic alteration plays an important role in nickel-induced carcinogenesis. Multiple epigenetic mechanisms have been identified to mediate nickel-induced gene silencing. Nickel ion is able to induce heterochromatinization by binding to DNA-histone complexes and initiating chromatin condensation. The enzymes required for establishing or removing epigenetic marks can be targeted by nickel, leading to altered DNA methylation and histone modification landscapes. The current review will focus on the epigenetic changes that contribute to nickel-induced gene silencing.

## 1. Introduction

Nickel (Ni) is one of the most abundant elements in our planet. Ni compounds are extensively used in numerous industrial processes, including the production of coins, jewelry, stainless steel, medical devices, Ni-Cd batteries, and Ni refinery, *etc*. Occupational exposure to Ni compounds through inhalation has become a big health concern for workers involved in the different stages of Ni processing [1,2]. Epidemiological studies have reported an increased incidence of lung and nasal cancer among nickel refinery workers [3,4,5,6]. It is worth noting that almost all nickel refinery workers smoked cigarettes, suggesting a possible synergistic effect of insoluble nickel compounds and cigarette smoking in cancer induction. In addition, insoluble chronic exposure to Ni compounds induced tumor formation in various animal models, further supporting the carcinogenic activity of nickel compounds [7,8,9]. Moreover, both water-soluble and insoluble compounds can transform cultured mammalian cells *in vitro* [10,11,12,13]. In 1990, the International Agency for Research on Cancer (IARC) classified all Ni (II) compounds as known human carcinogens (group 1) and metallic nickel as possible human carcinogens (group 2b) [13,14,15].

Multiple mechanisms have been proposed to mediate Ni-induced carcinogenesis, including its genotoxic and mutagenic activity, hypoxia-mimicking effects, dysregulation of cell signaling, and alterations of the epigenetic landscape [16,17,18]. DNA strand breaks and chromosomal aberration have been observed in Ni-exposed cells [19]. Compared to active euchromatic regions, transcriptionally inactive heterochromatic regions are more susceptible targets of Ni-induced damage. An early study from our laboratory demonstrated that Ni compounds selectively damage the heterochromatic long arm of the X chromosome in Chinese hamster ovary (CHO) cells [20]. The insoluble nickel compounds green nickel oxide and crystalline nickel monosulfide induce amplification of the ect-2 proto-oncogene in C3H/10T1/2 Cl 8 mouse embryo fibroblasts, which is found in green NiO and crystalline NiS-transformed 10T1/2 cells. Hence, the genotoxic activity of insoluble nickel compounds, in terms of gene amplification, is likely part of the molecular mechanisms by which nickel compounds induce morphological and neoplastic cell transformation [21]. In addition to its genotoxicity, Ni compounds are able to stabilize hypoxia inducible factors (HIFs) by inhibiting the activity of the HIF prolyl- and asparaginyl-hydroxylases [22,23]. Ni induced HIFs stabilization activates HIF-dependent transcription and initiates a series of hypoxia-specific responses under normoxia condition [24]. As a common phenomenon in many solid tumors, hypoxia arises in rapidly growing tumors due to the limitation of oxygen diffusion, and further facilitates tumor progression and metastasis. Thus, the hypoxia-mimicking effect of Ni compounds has been considered as an important mechanism underlying Ni-induced carcinogenesis [16]. 

Recent advances in cancer research have shown that epigenetic alterations play an important role in tumor formation and progression [25,26]. In the past two decades, research conducted in our laboratory and others have established the role of Ni in modulating the epigenetic landscape [16,27]. Ni is able to target the epigenetic machinery and induce alterations in chromatin structure, DNA methylation and histone modifications. 

## 2. Epigenetics and Gene Silencing

Epigenetics refers to the reversible but inheritable changes in gene expression that occur without alterations in DNA sequence [28]. In eukaryotes, genomic DNA is tightly packed with histone proteins to form a highly dynamic structure called chromatin. Depending on the degree of DNA condensation, chromatin can be divided into two different forms: heterochromatin (closed form, highly condensed region with little or no transcriptional activity) and euchromatin (open form, less condensed region with more active transcription). These “open” and “closed” chromatin domains are important for the accessibility of genetic information and have significant impacts in chromatin related biological functions, such as DNA replication, DNA recombination, DNA repair, transcription activation and repression, transcription initiation, elongation and termination, *etc*. [28].

The basic structural unit of chromatin is the nucleosome, which is composed of ~147-bp double strand DNA wrapped around an octamer core with two sets of histone proteins H2A, H2B, H3 and H4. The N-terminal tail of each histone protein protrudes from the nucleosome and serves as a platform for various chemical modifications. The histone modifications cooperate with other epigenetic factors including DNA methylation and small non-coding RNAs, not only influencing local chromatin structure but also playing an important role in DNA accessibility and gene expression. 

### 2.1. Histone Modification

The N-terminal tails of four histone proteins can be modified by various post-translational modifications at more than 60 different residues. While lysine residues can be modified by acetylation, methylation, sumoylation, ubiquitination and biotinylation, arginine can be modified by methylation, citrullination and ADP-ribosylation. Other modifications include phosphorylation of serine and threonine, ADP-ribosylation of glutamic acid, and cis-trans isomerization of proline [29]. Depending on the position and the protein bound, the modifications may have different functions on chromatin remodeling and gene expression [29].

Among these modifications, lysine acetylation and methylation have been extensively studied. Acetylation of lysine residues removes the positive charge from their side chains and decreases the affinity between histone tails and DNA, which subsequently leads to increased DNA accessibility and transcription activation in the promoter region of genes. Acetyl groups can be transferred to lysine residues by histone acetyltransferases (HATs) and removed by histone deacetylases (HDAC). These enzymes are normally associated with different large multi-protein complexes to either activate (HATs) or repress (HDACs) gene transcription [29,30]. 

In addition to acetylation, lysine residues can be mono-, di- or tri-methylated. Unlike acetylation, methylation of lysine does not change the positive charge on the side chain [29]. Therefore, depending on the position of the lysine residue and the number of methyl groups, lysine methylation can be associated with either active or repressive transcription. For example, di- and tri- methylation of histone H3 lysine 9 (H3K9) are repressive marks. While tri-methylated H3K9 (H3K9me3) is associated with heterochromatic regions, di-methylated H3K9 (H3K9me2) is found mostly in euchromatic regions and linked to the promoter of silenced genes. Other well-studied methylation marks include methylated H3K4 (active marks), tri-methylated H3K27 (a repressive mark) and methylation of H3K36 (a mark associated with transcription elongation) [29,31]. 

Similar to histone acetylation, lysine methylation is tightly controlled by a number of methyltransferases and demethylases. Most lysine methyltransferases (KMTs) contains a Set domain, and use S-adenosyl methionine (SAM) as the methyl donor. Two major types of lysine demethylases (KDMs) were identified. Lysine-specific demethylase 1(LSD1) is a flavin-dependent monoamine oxidase, which removes mono- or di-methyl groups from H3K4 and H3K9 using FAD as a co-factor. Another type of lysine demethylase belongs to a big family of jmjc-domain containing dioxygenases that have more than 27 family members. Jmjc-doamin containing demethylases require oxygen, 2-oxoglutarate and iron as co-factors, and are able to remove mono-, di-, and tri-methyl groups from many different lysine residues [31].

### 2.2. DNA Methylation

DNA methylation is a covalent modification in which a methyl group is added to 5-position of cytosine. In mammalian cells, the majority of 5-methylcytosine (5mC) is located in the context of CpG dinucleotides. About 60–90% CpGs are methylated in mammalian cells, and the methylation pattern correlates with long term transcriptional silencing, such as genomic imprinting, X-chromosome inactivation, suppression of repetitive elements, as well as maintaining lineage specific gene silencing. It is worth noting that, while DNA methylation in the promoter is typically linked to transcription repression, gene body methylation is more likely correlated to transcription activation [32]. 

Due to its essential role in maintaining genomic stability and modulating gene expression, 5mC levels are dynamically regulated by DNA methylation and demethylation [33]. First, cytosine methylation is catalyzed by DNA methyltransferases (DNMTs), a family of enzymes that is able to transfer the methyl group from SAM to cytosine. While DNMT3A and DNMT3B establish 5mC patterns in germ cells and developing embryos via de novo DNA methylation, DNMT1 and its partner (nuclear protein 95) specifically bind to hemimethylated DNA during DNA replication and copy 5mC marks from the parental strand to the newly synthesized strand [34]. Second, methyl groups can be gradually removed from cytosines across generations by lack of DNA methyltransferases or their activities during DNA replication. This process is called passive DNA demethylation and it depends on cell division and contributes to the global demethylation in the maternal pronuclei of the zygote. Lastly, recent advances have identified an active DNA demethylation process that involves the ten-eleven translocation (TET) methylcytosine dioxygenase, activation-induced cytidine deaminase (AID) and thymine DNA glycosylase (TDG) [35]. During active demethylation, 5mC is first oxidized by TET proteins using iron, oxygen and 2-oxoglutarate as co-factors. This reaction converts 5mC to 5-hydroxymethylcytosine (5hmC), 5-formylcytosine (5fC), and 5-carboxylcytosine (5caC), which can be subsequently removed by AID or TDG and base excision repair system [35]. Active demethylation mechanism has been implicated in reprogramming of the DNA methylation profile in primordial germ cells and the paternal pronuclei of the zygote. 

How does DNA methylation suppress gene transcription? DNA methylation in gene promoter regions may interfere with the binding of transcription factors that are required for gene activation. Long-term gene silencing may be mediated by a group of proteins that contain a methyl-binding domain (MBD). MBD proteins recruit co-repressors such as HDACs, and other chromatin modifing enzymes to form compact and repressive chromatin. There are five MBD proteins, MeCP2, MBD1, MBD2, MBD3 and MBD4, and each MBD protein recruits different sets of protein partners and represses different target genes. Recent genome-wide studies confirmed the correlation between DNA methylation and chromatin accessibility [32]. 

## 3. Nickel and Gene Silencing

Evidence of Ni-induced gene silencing first emerged toward the end of the 1980s. Conway and Costa [19,20] reported that the incidence of nickel-induced transformation is about 2–3-fold higher in male Chinese Hamster Embryonic (CHE) cells than those in female CHE cells, while there was no gender difference in 3-methylcholanthrene (MCA) induced CHE transformation. Karyotype analysis revealed that the majority of nickel-transformed cells exhibited either deletion or translocation of the long arm of the X chromosome (Xq), suggesting a possible role of the X chromosome in Ni-induced cell transformation of CHE cells. Interestingly, re-introducing the normal Chinese Hamster X chromosome into nickel-transformed cells resulted in cell senescence [36], indicating inactivation of one or several unknown genes located on the X chromosome as a prerequisite for Ni-induced transformation of CHE cells. 

The ability of nickel to silence gene expression was soon confirmed using a transgene system in which the endogenous hypoxanthine-guanine phosphoribosyltransferase (*HPRT*) gene in Chinese Hamster V79 cells was mutated by UV light and replaced by a small bacteria xanthine-guanine phosphoribosyl transferase (*gpt*) gene [37]. The cells that carry the *gpt* transgene can be selected by the resistance to either 6-thioguanine (6TG) for *gpt* inactive cells or HAT (hypoxanthine, aminopterin, and thymidine) medium for *gpt* active cells. One of the transgene cell lines, G12, has the *gpt* gene inserted near the telomere region of chromosome 1 which is close to a long stretch of heterochromatin. G12 cells exhibit a high level of 6TG resistance after exposure to water-insoluble Ni compounds (nickel sulfide, nickel subsulfide, and nickel oxides) or long term exposure to a water-insoluble Ni compound (nickel chloride), indicating Ni exposure is able to silence the *gpt* transgene [38,39]. Interestingly, Ni-induced *gpt* gene silencing can also be observed in an additional transgenic cell line, G10, however, *gpt* silencing in G10 cells was much less efficient compared to that in G12 cells. The difference between G12 and G10 is that *gpt* gene was inserted near a euchromatic region of chromosome 6 in G10 cells [39], indicating that the location of the transgene is critical for Ni-induced gene silencing.

In addition to transgene, several endogenous genes were silenced in C3H/10T1/2 mouse embryo cells transformed by green NiO and crystalline NiS, including the vitamin D receptor interacting protein 80 (DRIP80) gene, the insulin-like growth factor 1 (IGFR1) gene, the small nuclear activating protein C3 (SNAP C3) gene, the b-centaurin-2 gene and FAD synthetase gene [40,41,42].

### 3.1. Nickel and Heterochromatinization

**Figure 1:**
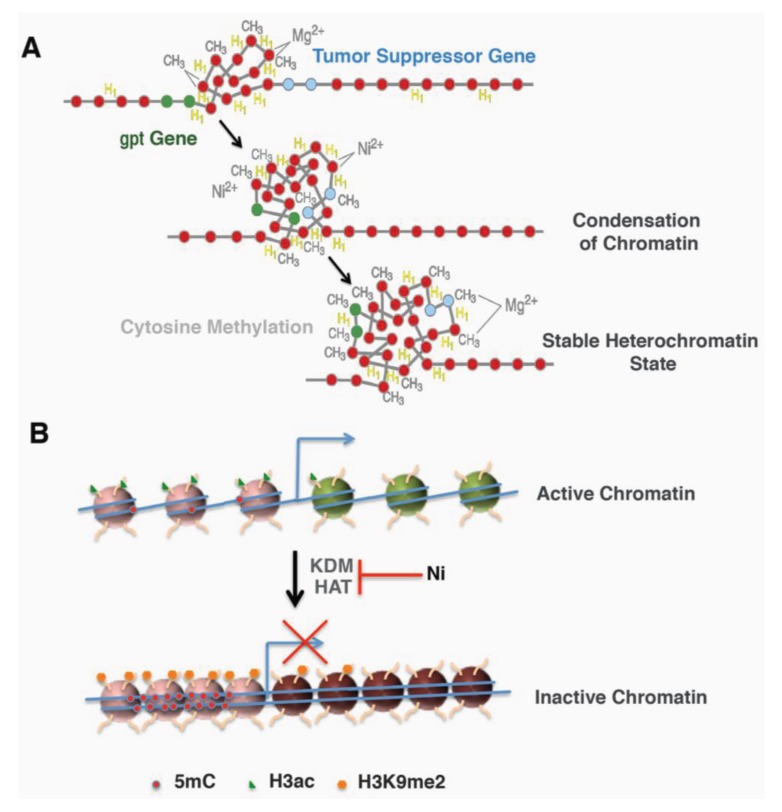
Schematic models for nickel mediated gene silencing. (**A**) Nickel increased local heterochromatinization and DNA methylation led to inactivation of nearby genes (Adapted from Ellen 2009 [44], @2009 American Chemical Society). (**B**) Transcription active genes exhibited an open active chromatin with less DNA methylation and more active histone mark histone H3 acetylation (H3ac). Nickel treatment converted it to an inactive chromatin by inducing repressive mark H3 lysine 9 dimethylation (H3K9me2) and DNA methylation (5mC) as well as inhibiting H3 acetylation.

Two approaches were designed to further address whether the location of the *gpt* transgene contributes to its sensitivity to Ni-induced silencing. First, heterochromatin fraction was isolated from G10, G12 and a 6-TG resistant clone derived from Ni-treated G12 cells and analyzed for the amount of *gpt* transgene. The amount of *gpt* transgene was highly enriched in the heterochromatin fraction from either G12 cells or G12-derived 6-TG resistant clone, but not in G10 cells. In Ni-treated G12 clones, the portion of the *gpt* gene associated with the heterochromatin fraction was significantly increased compared to those in parental G12 cells [43]. This result indicated that the heterochromatin location of the *gpt* gene in G12 cells not only contributes to its silencing induced by nickel, but also sensitizes the cells to Ni-induced silencing. The second approach used DNase I to probe the higher order of chromatin structure surrounding the *gpt* transgene in freshly isolated nuclei. A sensitive DNase I PCR method revealed an immediate DNA condensation in G12 nuclei following 1 h nickel treatment *in vitro*, which was not seen in G10 nuclei. Also, the sequence surrounding the *gpt* transgene exhibited a time-dependent increase in condensation following Ni treatment [43]. More interestingly, Ni-treated clones derived from G12 cells are more resistant to DNase I digestion [43]. These effects on G12 cells were inhibited by excess magnesium ion. These results indicate that nickel treatment is able to induce chromatin condensation near heterochromatic region, a process called heterochromatinization, which may inactivate nearby genes, such as the *gpt* transgene or tumor suppressor genes (Figure 1A).

Involvement of heterochromatinization in Ni-induced gene silencing was soon confirmed by reconstitution of nucleosomal array using atomic force microscopy [44]. In the same concentration, nickel-exposed oligonucleosomes exhibited a much greater condensation compared to those exposed to magnesium. Similar results were obtained by circular dichroism spectropolarimetry, indicating that nickel-treated oligonucleosomes form a more compact higher-order chromatin structure than magnesium-treated oligonucleosomes [44]. Moreover, nickel treatment enhanced the resistance of the *gpt* transgene to DNase I digestion in G12 cells (*gpt* transgene inserted near heterochromatin) but not in G10 cells (*gpt* transgene inserted in euchromatin). The resistance was not seen in G12 cells treated with cobalt and was much weaker in magnesium-treated cells [43]. This data, together with our earlier studies, indicates that Ni-mediated heterochromatinization plays an important role in Ni-induced gene silencing. 

### 3.2. Nickel and DNA Methylation

Other than heterochromatinization, DNA methylation is the first epigenetic factor found to contribute to Ni-induced gene silencing. 

In G12 cells, the *gpt* transgene silenced by nickel sulfide can be reactivated by treating cells with 5-aza-cytidine, a strong inhibitor of DNA methyltransferase. DNA hypermethylation was confirmed in the *gpt* transgene using methylation sensitive restriction enzymes [43]. Moreover, chromatin immunoprecipitation showed that methyl-binding protein MeCP2 was enriched at the *gpt* transgene in nickel silenced clones. This enrichment was not seen in parental G12 cells [45]. These results indicated that nickel exposure was able to induce local DNA methylation that subsequently silenced the *gpt* transgene. 

In addition to the *gpt* transgene, DNA methylation has been associated with Ni-induced silencing of endogenous genes in cultured cells. Nickel exposure of human bronchial epithelial (BEAS-2B) cells induced epithelial-mesenchymal transition (EMT). *E-cadherin*, which encodes a cell surface adhesion glycoprotein, was silenced in Ni-treated cells by DNA hypermethylation in its promoter region [46]. Similarly, O^6^-methylguanine DNA methyltransferase (MGMT), an enzyme that repairs O^6^-methylguanine, was silenced by promoter hypermethylation in NiS-transformed human bronchial epithelial (16HBE) cells [47]. Recent studies on the Syrian hamster (SHD) cell lines immortalized by soluble nickel demonstrated that hypermathylation of CpG cluster immediately upstream of p16 exon 1 resulted in p16 gene silencing and consequent cell senescence bypass [48]. 

Ni-induced promoter hypermethylation was also observed *in vivo*. Nickel sulfide was implanted into p53 heterozygous (p53+/−) mice to induce tumor formation. Malignant fibrous histiocytomas developed in both wild type and p53+/− mice, and all the tumors exhibited promoter hypermethylation of the tumor suppressor gene *p16* [49]. Moreover, intramuscular injection of nickel subsulfide to Wistar rats induced muscle tumors that displayed DNA hypermethylation in the promoter regions of *RARβ2*, *RASSF1A* and *p16* genes [50]. Thus, DNA hypermethylation and subsequent silencing of tumor suppressor genes may serve as an epigenetic mechanism mediating nickel’s carcinogenicity (Figure 1A). 

### 3.3. Nickel and Histone Modification

In addition to DNA methylation, local histone modification may also contribute to Ni-induced gene silencing. Histone acetylation is normally associated with gene activation. Exposure to Ni and Ni compounds reduced the global levels of acetylated lysine in all four histones in yeast, rat and human cells [51,52,53]. Nickel is able to inhibit histone H4 acetylation *in vitro*, suggesting that HAT activity may be one target of nickel [51,54]. *In vitro* activity assay confirmed a dose-dependent inhibition of HAT activity by nickel chloride [54]. Most interestingly, addition of the antioxidants in the reaction significantly reversed nickel induced inhibition of HAT activity, while co-treatment with hydrogen peroxide led to more inhibition [54]. It has been shown that nickel exposure increased reactive oxygen species (ROS) production and initiated the cell response to oxidative stress [55,56]. These results suggested a role of oxidative stress in nickel induced epigenetic changes. In addition, TSA, a potent HDAC inhibitor, is able to reduce the Ni-induced gene silencing in both yeast and mammalian cells [57], indicating that HDAC may be involved in Ni-induced gene silencing. Moreover, a decrease in H3 and H4 acetylation was observed in the promoter of the *gpt* transgene in G12 cells [45]. TSA, with or without 5-aza-cytidine, is able to reactivate *gpt* gene silenced by nickel. Furthermore, exposure of nickel-transformed cells to TSA significantly reduced the transformation phenotype induced by nickel, suggesting that gene silencing mediated by histone modification may play a role in nickel-induced cell transformation [58]. It is worth noting that a recent study has shown that Ni can bind histone H4 tails and form a structure similar to lysine acetylation, which might prevent the lysine residues from further acetylation [59].

Methylation of H3K9 is normally associated with gene silencing. Cells exposed to nickel exhibited a significant increase in global levels of dimethylated H3K9 (H3K9me2) [60]. Chromatin immunoprecipitation confirmed the increase of H3K9me2 in several gene-specific promoters, which likely contributes to the reduced expression of those genes [60,61]. Further studies demonstrated that the increase of H3K9me2 was due to nickel inhibiting the activity of jmjc-domain containing demethylases [60,62]. Jmjc domain, which is a key structural motif shared by the members of non-heme dioxygenase superfamily, contains a catalytic site that binds the iron. The affinity constant of Ni (II) binding to this iron-binding motif is about three times greater than that of iron. Therefore, nickel is more likely to replace the iron at the iron-binding site, which permanently inhibits the demethylase activity [62]. Spry2, a downstream target of the H3K9me2 demethylase JMJD1A, was silenced by chronic exposure to nickel, and this potentiated nickel-induced anchorage-independent growth [61]. 

The investigations described above allude to a possible mechanism of Ni-induced carcinogenesis (Figure 1B). Nickel is able to modulate histone acetylation and methylation by targeting the enzymes that add or remove the marks. The changed enzyme activity as well as the histone marks may lead to altered gene expression and eventually contribute to nickel-induced carcinogenesis. 

## 4. Beyond Chromatin: microRNAs and Ni Induced Gene Silencing

MicroRNAs (miRNAs) are endogenous small noncoding RNAs (18–25 nt) that negatively regulate gene expression in a sequence-specific manner [63,64]. These single-stranded RNAs bind to complementary sequences in 3' or 5' untranslated regions as well as coding regions of target mRNAs and result in mRNA degradation or inhibition of protein synthesis. According to the latest update of miRBase (version 19) [65], there are more than 2,000 unique mature miRNAs in the human genome, which are actively involved in many cellular processes including cell proliferation, differentiation, apoptosis, and metabolism [64]. Many studies have shown that miRNA profiles differ between normal and tumor tissues, and dysregulated miRNAs may play a crucial role in cancer initiation and progression. Because one mature miRNA is able to target multiple mRNAs due to binding of partially complementary regions, dysregulated miRNAs in cancer cells may lead to tremendous changes in gene expression.

Recent studies have reported that miRNAs may play a role in Ni-induced cell transformation and tumorigenesis. Expression of miR-222 was significantly up-regulated in rat rhabdomyosarcomas induced by an intramuscular injection of nickel subsulfide as well as in nickel-transformed 16HBE cells [66]. miR-222, which is able to target several important tumor suppressor genes including p27, p57 and PTEN, has been found to be increased in many human cancers. Both p27 and p57 are important negative regulators of the cell cycle. Thus, deregulated miR-222 and reduced expression of p27 and p57 may contribute to accelerated cell growth observed in Ni-induced tumors as well as transformed cells [66].

Similar to protein-coding genes, miRNA expression can be regulated by DNA methylation in their promoter regions. miR-152, a tumor suppressor microRNA targeting DNMT1, was significantly down-regulated in nickel sulfide-transformed 16HBE cells [67]. As a result, DNMT1 levels increased, which lead to elevated DNA methylation levels and enriched MeCP2 at the promoter of miR-152. Moreover, while ectopic expression of miR-152 in nickel sulfide-transformed cells inhibited cell proliferation, expressing anti-miR-152 in normal 16HBE cells resulted in increased cell proliferation and colony formation [67]. These results clearly demonstrate that down-regulation of miR-152 contributes to nickel sulfide-induced cell transformation.

## 5. Conclusions

In the past two decades, research aiming to characterize the carcinogenicity of Ni compounds have uncovered the epigenetic alteration induced by nickel exposure. Multiple lines of evidence have indicated the ability of nickel to perturb the epigenome. First, nickel has been shown to initiate chromatin condensation and to silence transgenes located near heterochromatin. In addition, nickel exposure has been associated with DNA hypermethylation and transcriptional repression of tumor suppressor genes both *in vitro* and *in vivo*. Moreover, nickel is able to modulate various histone modifications by targeting the enzymes that add or remove the specific marks. Lastly, nickel may interfere with microRNA network to degrade mRNA or block protein synthesis. These changes in DNA methylation, histone modification and miRNA network, along with condensed chromatin structure, create an aberrant epigenetic landscape and contribute to tumor initiation and progression.
